# Case Report: Preliminary evaluation of time-resolved imaging of contrast kinetics magnetic resonance imaging for assessing temporal enhancement patterns in small animal head and neck tumors

**DOI:** 10.3389/fvets.2026.1785784

**Published:** 2026-05-07

**Authors:** Sunghwa Hong, Soyeon Kim, Eunji Kim, Junghee Yoon, Jihye Choi

**Affiliations:** Department of Veterinary Medical Imaging, College of Veterinary Medicine, Seoul National University, Seoul, Republic of Korea

**Keywords:** canine, case report, feline, radiation therapy, time resolved MRA, tricks

## Abstract

Time-resolved magnetic resonance angiography provides dynamic information on contrast passage reflecting tumor vascularity and enhancement kinetics. Time-Resolved Imaging of Contrast KineticS (TRICKS) is established in veterinary medicine for vascular mapping; however, its specific utility for evaluating intratumoral enhancement heterogeneity and treatment response has not been described. This case series aimed to evaluate the feasibility of TRICKS for assessing temporal enhancement patterns and semi-quantitative temporal indices in small animal head and neck tumors undergoing radiation therapy. Three client-owned animals (two dogs with intracranial meningiomas and one cat with ceruminous gland adenocarcinoma) underwent MRI including TRICKS, as well as triple-phase computed tomography, for diagnostic evaluation, radiation therapy planning, or post-treatment follow-up. Across all cases, TRICKS provided temporally resolved enhancement information that was not appreciable on conventional contrast-enhanced T1-weighted MRI or triple-phase CT. TRICKS delineated delayed peripheral filling, inward-progressive enhancement, and intratumoral temporal heterogeneity, enabling clearer differentiation of rapidly enhancing regions from delayed or suspected non-enhancing components. Semi-quantitative parameters (enhancement integral, mean time to enhance, maximum slope of increase, and mean slope of decrease) demonstrated regional differences consistent with these enhancement patterns. In two cases, TRICKS identified post-radiation changes and delineated non-enhancing parenchymal defects chronologically prior to their clear appearance on other modalities. These exploratory findings suggests TRICKS can provide time-resolved enhancement surrogates in veterinary patient tumors, contributing to improved characterization of tumor compartmentalization and post-treatment evaluation. However, this serves as a preliminary technical feasibility report, and further studies with larger cohorts and histopathological validation are warranted to validate these observations.

## 1 Introduction

Radiation therapy (RT) is widely used for the treatment of canine and feline head and neck tumors due to advances in treatment planning systems and the increased availability of linear accelerators in veterinary medicine. RT is applied as a primary or adjuvant modality to improve local tumor control and preserve normal organ function when surgical excision is limited by anatomical complexity or functional morbidity (1–3). Accurate assessment of treatment response is essential for prognostication and adaptive RT planning. In parallel with these clinical advancements, the role of animal models has been increasingly emphasized as a vital tool in medical research and the development of complex surgical and implant procedures (4, 5). Furthermore, the integration of artificial intelligence is beginning to redefine the landscape of veterinary medicine, particularly by enhancing diagnostic imaging and anatomical assessment (6, 7). These broad technological evolutions underscore the need for more sophisticated, time-resolved imaging techniques that can provide precise functional and anatomical insights during the course of treatment.

Conventional response evaluation methods, including the Response Evaluation Criteria in Solid Tumors (RECIST), primarily rely on changes in tumor size. However, because morphological changes often occur weeks after treatment, these criteria have limited sensitivity for detecting early biological response. As a result, there is increasing interest in functional imaging techniques that assess tumor vascularity and perfusion as early indicators of therapeutic efficacy.

Computed tomography (CT) is routinely used for RT planning and provides high spatial resolution. However, triple-phase CT depicts tumor enhancement at only a few discrete time points, limiting evaluation of dynamic intratumoral vascular behavior.

Time-resolved magnetic resonance angiography (MRA) is a dynamic contrast-enhanced magnetic resonance imaging (MRI) technique that acquires multiple phases during contrast passage with high temporal resolution (8–10). In veterinary patients, Time-Resolved Imaging of Contrast KineticS (TRICKS) has primarily been used for vascular mapping and visualization of tumor-associated vessels (11–13). However, its application for evaluating temporal enhancement characteristics within tumor parenchyma has not been investigated, despite the potential for time-dependent signal intensity changes to reflect intratumoral heterogeneity not appreciable on static contrast-enhanced T1-weighted (CE-T1W) MRI.

Semi-quantitative parameters derived from TRICKS, including enhancement integral (EI), mean time to enhance (MTE), maximum slope of increase (MSI), and mean slope of decrease (MSD), offer a practical means of assessing contrast kinetics (14–16). However, in this specific veterinary context, these parameters are non-normalized, signal-intensity-derived indices and remain unvalidated against reference standard perfusion techniques (e.g., dynamic susceptibility contrast MRI or CT perfusion). Therefore, interpreting these indices directly as absolute measures of tumor perfusion, vascular permeability, or cellular viability remains speculative without histopathological confirmation.

This case report aimed to describe the preliminary application of TRICKS in three small animal patients with head and neck tumors. The objective was to explore intratumoral enhancement heterogeneity and temporal enhancement patterns not fully captured by conventional CT or CE-T1W MRI. Given the small sample size and profound heterogeneity of the cases, this study serves as a descriptive, hypothesis-generating technical feasibility report rather than a definitive clinical validation.

## 2 Method

In this retrospective descriptive case series, three client-owned animals (two dogs and one cat) with head or neck tumors that underwent RT to the Seoul National University Veterinary Medical Teaching Hospital were included. All three animals underwent triple-phase CT (arterial, venous, delayed) and MRI including TRICKS, as a part of RT planning or post-treatment evaluation, and semi-quantitative temporal indices analysis was performed using TRICKS images. Informed owner consent was obtained prior to imaging and study inclusion. After placing the animal in ventral recumbency under general anesthesia, MRI examinations were performed on a 1.5-T system (SIGNA Creator; GE Healthcare, WI, USA) with an 8-channel phased array head or neck coil appropriate for animal size. The imaging protocol included T2-weighted (T2W) images, pre T1-weighted (pre-T1W) images, CE-T1W images, and TRICKS sequences covering the entire tumor region. TRICKS sequences were obtained after intravenous administration of 0.2 mmol/kg of a gadolinium-based contrast agent (Dotarem; Guerbet, France) at a rate of 2 mL/s using an automatic power injector (OptiStar^®^ Elite; Mallinckrodt, MO, USA) through a cephalic vein catheter, followed by a saline flush. The TRICKS sequence was acquired using a dorsal imaging plane with a repetition time of 4–5 ms and an echo time of 1–2 ms. The flip angle was set to either 25° or 50°, and the field of view was approximately 280 mm. The matrix size was 320 × 224 or 320 × 256, corresponding to an in-plane pixel size of 0.9 × 1.1 or 0.9 × 1.2 mm. Slice thickness was 1.8 or 2.0 mm, and number of excitations was 1. Baseline mask images were obtained first, and dynamic post-contrast TRICKS acquisition began immediately with minimal scan delay. Following the acquisition, baseline-subtracted images were reconstructed. Two-dimensional reconstructed MRA images were generated using maximum intensity projection, and the dynamic time-phase images were concatenated into a single cine series. TRICKS images were analyzed using ReadyView (GE Advantage Workstation, Palo Alto, CA, USA). Semi-quantitative temporal parameters, including EI, MTE, MSI, and MSD, were automatically calculated from time signal intensity curves (TICs) generated. EI was calculated as the cumulative signal intensity above the pre-contrast baseline across all postcontrast phases, representing the area under the enhancement curve, and expressed in arbitrary units because it depends on signal intensity, which has no absolute unit (17, 18). MTE represents the mean time required for the contrast bolus to reach peak enhancement within a given tissue region during the first pass phase and was recorded in seconds. MSI was obtained by identifying the steepest positive increase in signal intensity between consecutive post-contrast phases, reflecting the rapid first-pass wash-in related to lesion vascularity. MSD was obtained as the greatest decline in signal intensity between successive phases, representing the wash-out behavior associated with vascular permeability and extracellular leakage. MSI and MSD values were reported in arbitrary units because they were derived without normalization to time (19, 20). These indices were derived from manually placed regions of interest (ROIs) based on visual assessment of enhancement patterns on TRICKS images, which introduces inherent subjectivity and potential interobserver variability. Furthermore, these metrics are signal-intensity-derived temporal indices and are not absolute measures of tissue perfusion, as they were not cross-validated against a gold-standard perfusion technique (e.g., dynamic susceptibility contrast MRI or CT perfusion).

## 3 Results

### 3.1 Case 1

**Figure 1:**
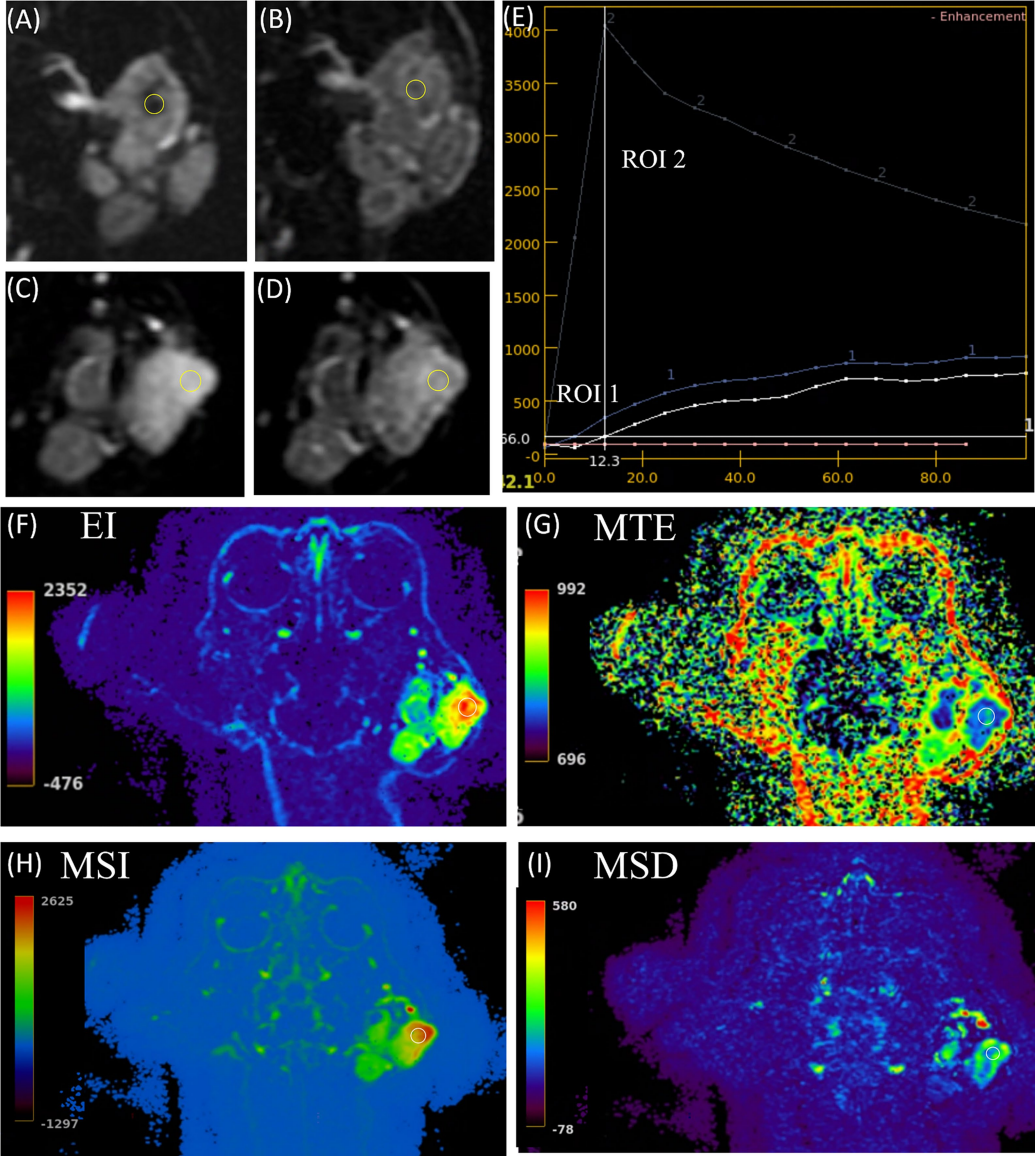
The contrast enhancement changes and semi-quantitative temporal parameter color maps of the tumor’s central low-enhancing area and contrast-enhancement patterns over time in a 14-year-old spayed female Persian cat with ceruminous gland adenocarcinoma (Case 1) evaluated using time-resolved imaging of contrast kinetics (TRICKS). **(A,B)** Dorsal images from the 3rd and 16th phases of TRICKS, respectively, demonstrate clearer delineation of the suspected non-enhancing region (yellow circle) compared to triple-phase CT, with dynamic and continuous changes in contrast enhancement visible over time (not shown). In the 16th phase **(B)**, rim-like enhancement developed around the central low-enhancing area, exhibiting signal intensity higher than that of the adjacent tumor parenchyma. Semi-quantitative analysis demonstrated a time to peak ≥ 68 s in the central region (yellow circle), markedly longer than that of the surrounding tissue (12 s). **(C,D)** Dorsal images from the 3rd and 7th phases of TRICKS show dynamic enhancement depending on tumor location. The most lateral mass (*) demonstrated markedly higher signal intensity than the surrounding masses during the 3rd phase **(C)**, creating a pronounced contrast between them. In the 7th phase **(D)**, however, the signal intensity of the lateral mass decreased, resulting in a reduced difference compared with the surrounding masses. In contrast, triple-phase CT demonstrated relatively similar degrees of contrast enhancement among the surrounding phases (not shown). **(E)** Time–intensity curves (TICs) for two regions: the area marked with a yellow arrow in **A,B** (region of interest [ROI] 1) and the area marked with an asterisk in **C,D** (ROI 2). In the TIC, ROI 1 shows a very shallow slope with a slow rise to a relatively low maximum, whereas ROI 2 reaches its peak at the 3rd phase and then declines relatively rapidly. **(F–I)** Color maps derived from the asterisk-marked region in **C,D** are shown: enhancement integral (EI, **F**), mean time to enhance (MTE, **G**), maximum slope of increase (MSI, **H**), and maximum slope of decrease (MSD, **I**). The corresponding area demonstrates high values on both the EI (**F**) and MSI (**H**) maps, low values on the MTE (**G**) map, and mixed high and low values on the MSD (**I**) map.

A 13-year-old spayed female Persian cat (3.1 kg) presented with a mass of the left ear accompanied by pruritus, fever, and bleeding. On admission, temperature was 39.4 °C, heart rate 132 beats/min, respiratory rate 60 breaths/min, and blood pressure 136/115 mmHg (mean 120 mmHg). Incisional biopsy confirmed ceruminous gland adenocarcinoma. CT and MRI revealed a left ear canal mass occupying the entire lumen. It infiltrated beyond the ear canal along the ventral canal wall into the mandibular region and parotid regions. It was measured approximately 3.2 × 3.4 × 3.9 cm. On MRI, the mass was isointense on pre-T1W images and hyperintense on T2W images, with marked heterogeneous enhancement following contrast administration. On triple-phase CT images, a centrally located non-enhancing area was observed, and only faintly discernible enhancement changes was seen during the arterial and delayed phases. This area was considered suspicious for intratumoral necrosis. When contrast-enhanced MRI was reviewed, the corresponding region showed only a focal area of intense enhancement with limited delineation of its internal components, and necrosis could not be confirmed on MRI alone. TRICKS provided detailed temporal information that was not appreciable on CT or static MRI (Figure 1). During early TRICKS phases, a portion of the mass appeared cystic, followed by progressive contrast enhancement filling of this region in the later phases. Rim-like enhancement surrounding the central region was apparently visualized on TRICKS. Intratumoral and peritumoral vessels were clearly depicted on maximum intensity projection images reconstructed from TRICKS, whereas these structures were not identifiable on CE-T1W MRI. TRICKS also demonstrated temporal enhancement differences among various intratumoral subregions: the most lateral regions showed markedly higher signal intensity during early phases and more conspicuous time-dependent enhancement. In contrast, these differences were less distinct on CE-T1W images as well as all triple-phase CT phases. TIC graphs were generated by placing two regions of interest (ROIs) based on the distinct temporal enhancement patterns, observed on TRICKS. The region that appeared cystic during the early TRICKS phases and demonstrated progressive contrast filling in the later phases was designated as ROI 1. In contrast, an intratumoral subregion that showed markedly stronger and more conspicuous time-dependent enhancement was designated as ROI 2; these enhancement differences were only mildly appreciable on CE-T1W images and were indistinct across all triple-phase CT phases. The obtained graphs showed clearly distinct enhancement patterns between the two regions. ROI 1 displayed a gradual, delayed enhancement profile, whereas ROI 2 exhibited a steep early rise. Semi-quantitative temporal parameters derived from TRICKS supported these findings. The highly vascular region showed higher EI and MSI values, lower MTE, and heterogeneous MSD distribution compared with the surrounding areas, reflecting rapid contrast wash-in (Figure 1). Based on the pre-treatment imaging findings, the mass extensively involved the left ear canal with invasion into the adjacent mandibular and parotid regions and demonstrated a central non-enhancing component. Palliative radiotherapy was elected because complete surgical excision was infeasible due to extensive local invasion and the tumor continued progression despite chemotherapy with toceranib administered at a dose of 2.5 mg/kg. RT was delivered using volumetric modulated arc therapy (VMAT) at 6 Gy × 6 fractions, once weekly. Post-treatment CT and MRI showed a marked reduction in tumor size with improved ear canal patency. No recurrence was observed during follow-up imaging over more than 7 months.

### 3.2 Case 2

**Figure 2:**
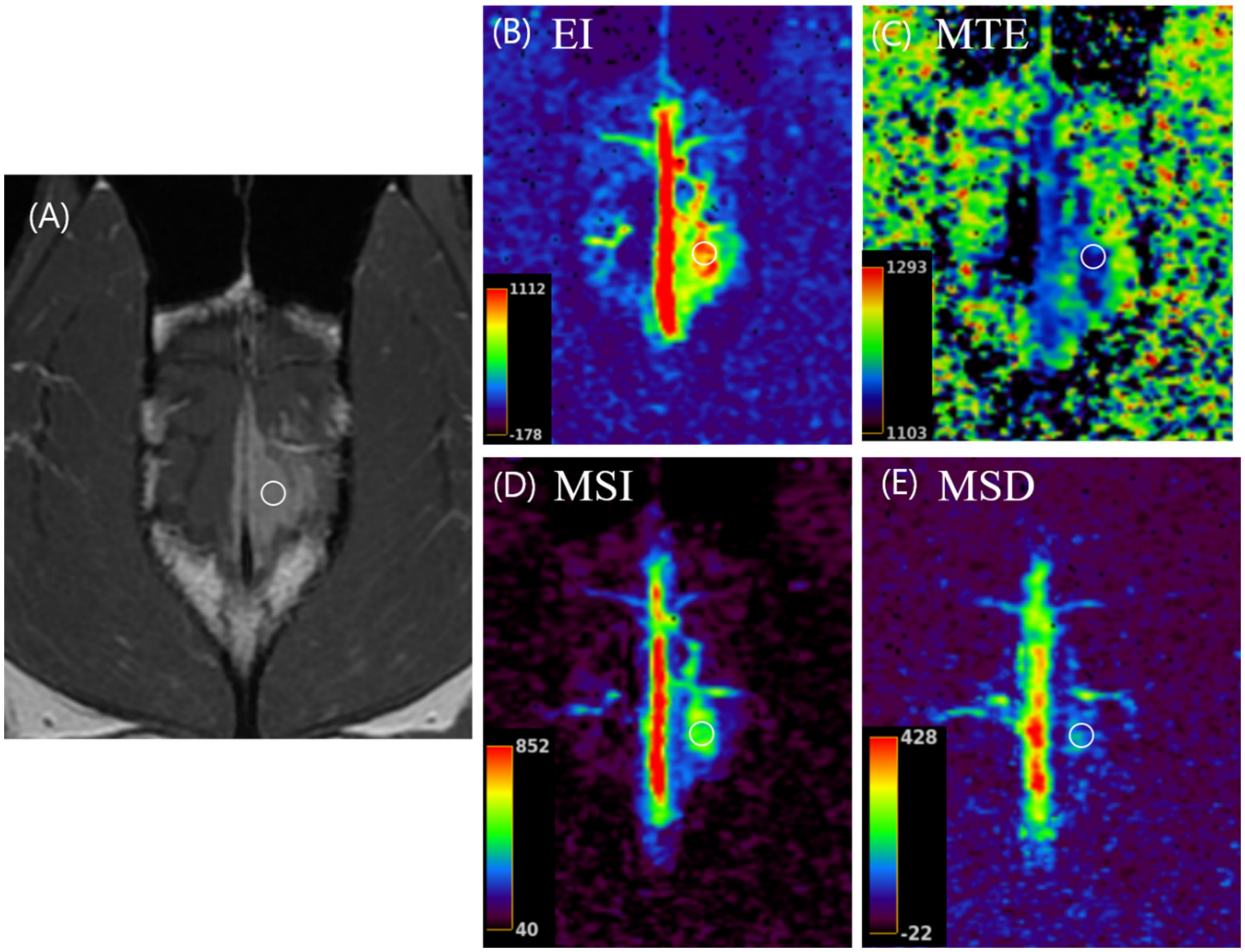
Post-contrast T1-weighted magnetic resonance image (MRI) **(A)** and semiquantitative temporal parameter color maps—enhancement integral (EI, **B**), mean time to enhance (MTE, **C**), maximum slope of increase (MSI, D), and maximum slope of decrease (MSD, **E**)—in a 13.6-year-old spayed female Bull Terrier with a meningioma (Case 2). In the post-contrast T1-weighted image, a region of the tumor (white circle) shows moderately intense enhancement. The corresponding area appears with a high value on both the EI **(B)** and MSI **(D)** maps, a low value on the MTE **(C)** map, and mixed high and low values on the MSD **(E)** map.

A 13-year-old spayed female Bull Terrier (19.8 kg) was presented with a one-month history of recurrent seizures and ataxic gait. On physical examination, body temperature was 37.7 °C, heart rate 90 beats/min, respiratory rate 24 breaths/min, and systolic/diastolic blood pressure 145/70 mmHg. Based on the neurological signs, an intracranial neoplastic lesion was suspected. MRI revealed a broad, plaque-like extra-axial brain mass along the falx cerebri, extending into the parietal and occipital lobes. The lesion exhibited marked contrast enhancement, distinct dural tail sign, and focal intralesional hemorrhage, findings consistent with an extra-axial tumor. On conventional CE-T1W images, the mass appeared uniformly enhancing. Similarly, on triple-phase CT, the mass demonstrated a comparable contrast enhancement pattern, with only subtle changes between the arterial and delayed phases that were insufficient to clearly characterize intratumoral vascular heterogeneity. In contrast, TRICKS showed temporal changes in tumor enhancement kinetics. A portion of the tumor adjacent to the falx cerebri showed no enhancement during the early phases, followed by progressive and distinct enhancement in later phases, clearly demonstrating temporal heterogeneity within the lesion. Correspondingly, some subregions within this area exhibited enhancement heterogeneity, with higher EI and MSI values and a lower MTE compared with the surrounding tumor tissue, suggesting relatively increased blood flow. While the MSD values were mildly increased within a limited subregion, suggesting localized variability in contrast wash-in and wash-out characteristics (Figure 2). Based on the clinical signs and pre-treatment imaging findings, the lesion was diagnosed as a presumptive falcine meningioma with heterogeneous enhancement characteristics. Definitive radiotherapy was subsequently conducted, with VMAT administered at a dose of 3.75 Gy per fraction for 12 fractions, resulting in a total dose of 45 Gy delivered once daily. On RT completion, no appreciable change in tumor size or enhancement pattern was observed on CE-T1W images. However, EI maps demonstrated a distinct change in intratumoral enhancement pattern, characterized by increased intratumoral heterogeneity with a patchy appearance compared with pre-treatment imaging. Follow-up MRI performed 1 month after RT revealed a slight reduction in tumor size. Despite these imaging findings, progressive clinical deterioration was observed, and the patient died 2 months after completion of RT. The overall survival time from the initial diagnosis was 86 days.

### 3.3 Case 3

**Figure 3:**
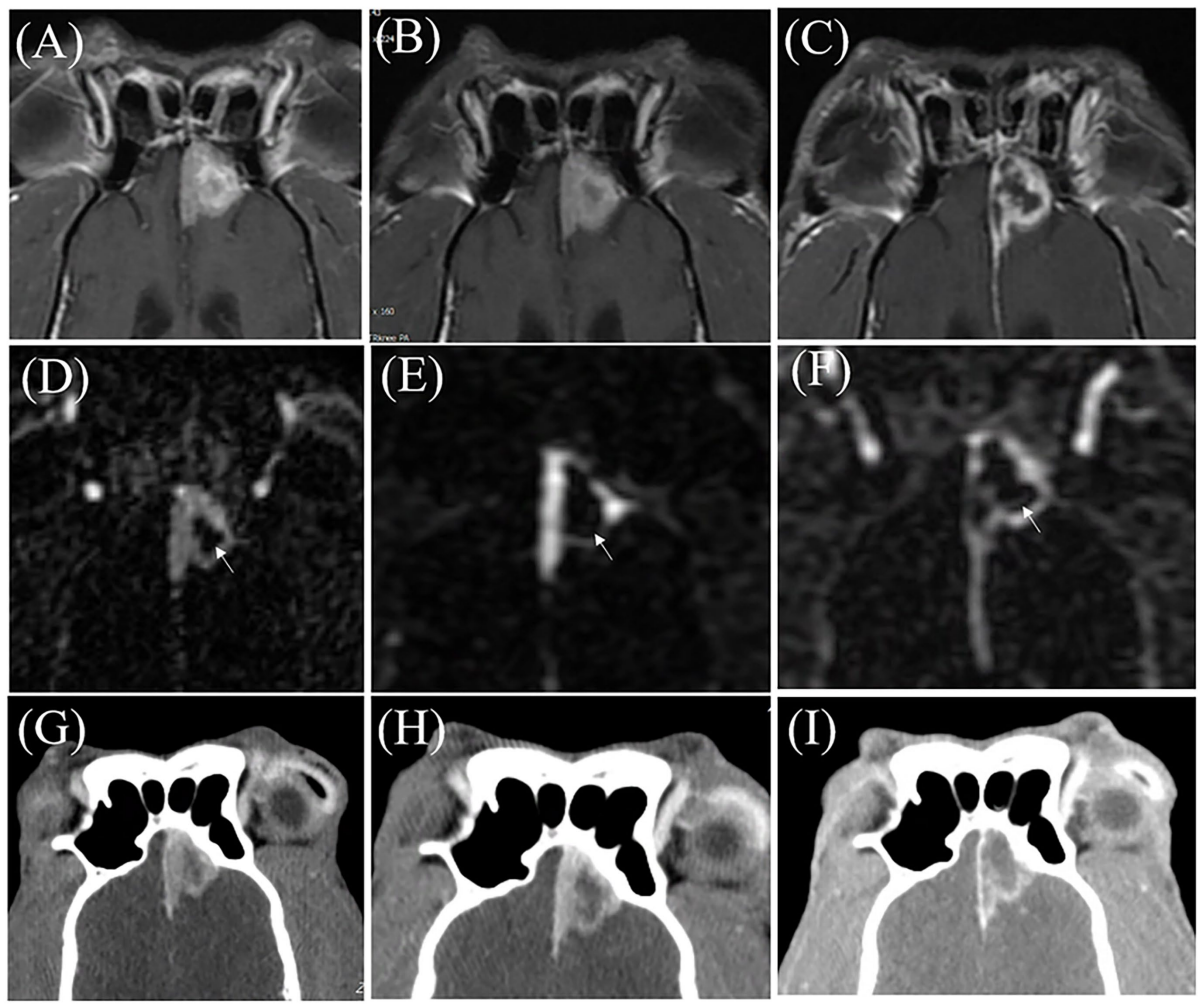
Follow-up imaging of an 11-year-old castrated male mixed-breed dog with meningioma (Case 3) at 1 month **(A,D,G)**, 3 months **(B,E,H)**, and 6 months **(C,F,I)** after completion of radiation therapy. **(A–C)** are contrast enhanced T1 weighted (CE-T1W) magnetic resonance images. **(D–F)** are images from the 25th phase of time-resolved imaging of contrast kinetics (TRICKS). **(G–I)** are delayed-phase of triple-phase computed tomography (CT) images. At 1-month follow-up **(A,D,G)**, TRICKS **(D)** reveals a broader area of non-enhancing parenchymal defect (arrow) within the tumor compared to CE-T1W **(A)** and triple-phase CT images **(G)**. Notably, CE-T1W image demonstrates more pronounced enhancement in regions that appear as non-enhancing defects on TRICKS. At 3-month follow-up **(B,E,H)**, the tumor appears similar in size and shape on CE-T1W **(B)** and triple-phase CT images **(H)**, while TRICKS **(E)** demonstrates further parenchymal thinning and an increased non-enhancing area (arrow). At 6-month follow-up **(C,F,I)**, all three modalities show a marked decrease in parenchymal enhancement.

An 11-year-old castrated male mixed-breed dog (7.4 kg) was presented with a history of seizure. On physical examination, body temperature was 38.7 °C, heart rate 124 beats/min, respiratory rate 42 breaths/min, and systolic blood pressure was 160 mmHg. On MRI, a solitary mass was found in the left frontal lobe, broadly attached to the falx cerebri and dorsal meninges. The mass contained cystic components and showed marked contrast enhancement with a distinct dural tail sign. These findings were consistent with an extra-axial brain tumor, most suggestive of meningioma. On conventional CE-T1W images, a focal portion of the tumor showed more intense enhancement compared with the surrounding tumor parenchyma. On triple-phase CT images, the corresponding region appeared slightly hypoattenuating compared with the surrounding tumor parenchyma, throughout the arterial, venous, and delayed phases, with minimal temporal variation, limiting assessment of its vascular characteristics. In contrast, TRICKS provided clearer temporal information. During the early TRICKS phase (6 phase), the region remained heterogeneously unenhanced, followed by progressive contrast filling and enhancement in the later phase (40th phase), indicating delayed contrast arrival of this intratumoral subregion. Semi-quantitative analysis supported these findings, as this region demonstrated higher EI values compared with surrounding areas, accompanied by mildly increased MTE and MSI values, consistent with relatively increased enhancement. MSD signals were generally low and indistinct, suggesting delayed washout and prolonged contrast retention. Based on the clinical signs and pre-treatment imaging findings, the lesion was diagnosed as a presumptive falcine meningioma with heterogeneous enhancement characteristics. D efinitive treatment was performed using stereotactic radiotherapy. VMAT was administered with a total dose of 24 Gy delivered in three consecutive daily fractions of 8 Gy each. On serial follow-up imaging performed at 1, 3, and 6 months after RT, TRICKS enabled more definitive visualization of non-enhancing parenchymal tissue through its superior temporal resolution. At the 1-month follow-up, TRICKS clearly identified a focal low enhancing region with well-defined margins, whereas the lesion appeared with ill-defined and heterogeneous enhancement on both triple-phase CT and CE-T1W images, making it difficult to characterize. However, by the 6-month follow-up, the morphology of the tissue of ill-defined enhancement tissue became consistently distinct and comparable across all modalities, including TRICKS, triple-phase CT, and CE-T1W (Figure 3). Therefore, TRICKS allowed for the visual delineation of progressive low-enhancing tissue chronologically prior to other imaging modalities. The patient has remained alive for more than one year following treatment.

## 4 Discussion

In all cases, TRICKS provided complementary functional information regarding delayed peripheral filling, inward-progressive enhancement, regional wash-in differences, and early depiction of the non-enhancing regions—features that were less conspicuous on conventional CE-T1W MRI or triple-phase CT. Although TRICKS did not alter the primary imaging diagnosis, it provided additional insight into intratumoral vascular behavior within a routine MRI examination.

The high temporal resolution of TRICKS enabled visualization of spatially heterogeneous enhancement patterns within tumor parenchyma. While static images may struggle to fully characterize these regions, time-resolved imaging demonstrated distinct temporal kinetics, such as delayed and gradual contrast accumulation versus rapid early enhancement. Accordingly, the characterization of these diverse temporal enhancement patterns may be better achieved using time-resolved imaging techniques rather than static post-contrast images alone.

TRICKS revealed distinct intratumoral enhancement kinetics in Cases 1 and 3, including regions with rapid early enhancement and others with delayed but progressive enhancement. These findings align with previous reports describing early- and delayed-enhancing tumor components (21, 22). In contrast, conventional CE-T1W MRI provided limited discriminatory information, as the corresponding delayed-enhancing or suspected non-enhancing regions appeared only mildly hyperintense or occasionally more prominent than the surrounding parenchyma, likely due to contrast diffusion during relatively long acquisition times. On triple-phase CT, hypoattenuating regions were visible during the arterial phase; however, clear delineation of the boundaries of these hypoattenuating regions or rim-like enhancement remained indistinct across phases. Unlike CT, which samples only a few discrete time points, TRICKS enables continuous temporal sampling, allowing clearer visualization of intratumoral compartmental differences based on contrast kinetics.

Temporal information obtained from TRICKS can be translated into semi-quantitative parametric maps reflecting regional signal-intensity kinetics, rather than absolute perfusion or vascular permeability. In Case 1, specific intratumoral subregions demonstrated higher EI and MSI values with lower MTE, indicative of rapid contrast wash-in. Similarly, in Case 3, focal subregions showed relatively elevated EI with mildly increased MTE and MSI compared with adjacent tissue, highlighting distinct temporal enhancement heterogeneity. In contrast, suspected non-enhancing regions exhibited low EI and MSI values, consistent with delayed or absent contrast arrival. However, as observed in Case 1, the intensely enhancing rim surrounding these regions suggests a complex compartmentalization. This underscores that TRICKS alone is insufficient for definitive tissue characterization; integration with additional functional imaging metrics, such as diffusion-weighted imaging, and direct histopathological validation are necessary to accurately delineate true biological tumor compartments (21, 22).

Following radiotherapy, TRICKS-derived enhancement maps demonstrated changes in intratumoral enhancement distribution that were not immediately accompanied by measurable tumor size reduction on conventional imaging. In Case 2, a marked post-treatment decrease in EI was observed within a previously rapidly enhancing subregion, resulting in values comparable to surrounding tumor tissue. While the exact biological correlates remain unverified, these changes may represent radiation-induced alterations in local contrast kinetics. Similarly, in Case 3, serial follow-up imaging demonstrated that TRICKS visually delineated progressive non-enhancing parenchymal defects chronologically prior to their clear appearance on static CE-T1W MRI or triple-phase CT. Collectively, these descriptive findings suggest that TRICKS may capture treatment-related alterations in temporal enhancement patterns, offering observational insights that supplement conventional morphological evaluation.

Several critical limitations should be acknowledged. First, the inclusion of only three cases representing different species, tumor histotypes, anatomical sites, and radiation protocols introduces profound heterogeneity, precluding any generalizable conclusions regarding clinical utility. Second, the parameters utilized are semi-quantitative, non-normalized indices that remain unvalidated against reference standard perfusion techniques in this specific veterinary context. Third, important sources of methodological variability were present: the manual placement of regions of interest was subjective and based on visual impression, acquisition parameters such as the flip angle differed between cases, and the reproducibility of these indices was not formally evaluated. Finally, it must be explicitly noted that the TRICKS findings did not alter the primary imaging diagnosis, clinical decision-making, or definitive radiation planning in any of these cases. Nevertheless, TRICKS offers the practical advantage of simultaneous visualization of peritumoral vasculature and temporal enhancement kinetics, serving as a valuable hypothesis-generating tool for future large-scale, prospectively validated studies.

In conclusion, TRICKS offers a practical method to visualize intratumoral enhancement heterogeneity and post-treatment temporal kinetics not fully captured by static imaging, although these exploratory findings serve strictly as a preliminary, hypothesis-generating technical feasibility report rather than evidence of clinical utility. Future large-scale prospective studies with histopathological validation are required to determine its true clinical value in veterinary oncology.

## Data Availability

The original contributions presented in the study are included in the article/supplementary material, further inquiries can be directed to the corresponding author.
